# Policing in Nonhuman Primates: Partial Interventions Serve a Prosocial Conflict Management Function in Rhesus Macaques

**DOI:** 10.1371/journal.pone.0077369

**Published:** 2013-10-22

**Authors:** Brianne A. Beisner, Brenda McCowan

**Affiliations:** 1 Department of Population Health & Reproduction, School of Veterinary Medicine, University of California Davis, Davis, California, United States of America; 2 California National Primate Research Center, University of California Davis, Davis, California, United States of America; Université Paris 13, France

## Abstract

Studies of prosocial policing in nonhuman societies traditionally focus on impartial interventions because of an underlying assumption that partial support implies a direct benefit to the intervener, thereby negating the potential for being prosocial in maintaining social stability for the benefit of the group. However, certain types of partial interventions have significant potential to be prosocial in controlling conflict, e.g. support of non-kin subordinates. Here, we propose a *policing support hypothesis* that some types of agonistic support serve a prosocial policing function that maintains group stability. Using seven large captive groups of rhesus macaques, we investigated the relationship between intervention type and group-level costs and benefits (rates of trauma, severe aggression, social relocation) and individual level costs and benefits (preferential sex-dyad targeting, dominance ambiguity reduction, access to mates, and return aggression). Our results show that impartial interventions and support of subordinate non-kin represent prosocial policing as both (1) were negatively associated with group-level rates of trauma and severe aggression, respectively, (2) showed no potential to confer individual dominance benefits, (3) when performed outside the mating season, they did not increase chances of mating with the beneficiary, and (4) were low-cost for the highest-ranking interveners. We recommend expanding the definition of ‘policing’ in nonhumans to include these ‘policing support interventions’.

## Introduction

Prosocial behavior is ubiquitous in humans, ranging from altruism and cooperation to punishment and policing [1]–[4], and the evolutionary roots of human prosociality can be traced by investigating similar behavior in nonhuman animals. Among our closest relatives, the primates, one particular type of prosocial behavior, i.e. policing, has been reported in a wide variety of species, ranging from the great apes [5]–[7] to macaques and baboons [8]–[11]. Among nonhuman primates, policing has been defined as impartial monitoring and attempted control of conflict among group members by third parties [8], [12]. Impartial means that the intervener shows no preferential treatment toward any conflict participant. In contrast, partial interventions involve support of one or the other conflict participant. Here, we re-evaluate this assumption of impartiality by investigating the potential for both partial and impartial interventions to function as policing in rhesus macaques.

### What is Policing?

Policing in animal societies generally refers to control of group conflict, be it impartial intervention to control group fighting, as in nohuman primates [8], killing worker eggs to control reproductive conflict, as in social insects [13], or general repression of competition, as in Frank’s model of the evolution of reproductive fairness among subunits, such as replicating units within a cell, individual insects within a colony, or humans within a society [14]. Within primatology, specifically, the concept of conflict control has been referred to as pacifying intervention [15], peaceful intervention [9], and impartial intervention [8], although earlier studies sometimes use these terms to describe any intervention to stop a fight, regardless whether the intervener’s goals appear to be selfish or prosocial. Notably, defining policing as being prosocial appears to be limited to the nonhuman primate literature, and only in recent years [8], [12]. It is most instructive, however, to consider the relative costs and benefits to the intervener as well as the group. Policing is essentially mutualistic behavior because both the policer and the group benefit from reduced group conflict. The question remains, however, whether the intervener gains additional benefits from policing. If so, natural selection may have favored policing behavior because policers gained these additional benefits, suggesting that policing may be functionally more similar to selfish behavior than to mutualistic or prosocial behavior. Given this framework, we address two questions regarding intervention behavior in a rhesus macaques: (1) Do impartial interventions function as prosocial policing? And (2) do some partial interventions function as prosocial policing?

### Benefits of Policing Beyond Reduced Societal Conflict

#### Impartial intervention

Among nonhuman primates, policing has been recently defined as impartial intervention [8], meaning a third party enters an on-going fight but shows no partial treatment for any conflict participant. This definition relies upon an implicit assumption that interveners who support one of the conflict opponents gain additional benefits, which negates the potential for the intervention to be prosocial in maintaining social stability for the benefit of the group. In order to satisfy this definition, impartial interventions must (a) yield a net benefit to the entire group, including the intervener, via reduced rates of conflict and (b) not confer any additional benefits to the intervener. A further consideration is cost – we expect that most policing behavior should be low cost, at least for the animals that most frequently perform these interventions, otherwise the behavior would not have evolved.

Impartial interventions in many primate societies do appear to meet these criteria. Impartial interventions by high-ranking individuals have been identified as a conflict management mechanism in pigtail macaques and chimpanzees [12], [16] because such interventions mitigate conflict among group members [17] and allow group members to build larger, more diverse social networks [18]. The cost of impartial interventions also appears to be low for high-ranking interveners such that the benefits gained likely outweigh the cost [8]. Impartial policing in rhesus macaques, however, has not previously been evaluated, perhaps because impartial interventions appear to be infrequent [19] or distribution of social power is assumed to be too uniform to permit even the highest-ranking animals to control the conflict of others [20]. Recently, McCowan and colleagues [21] argued that high-ranking animals do control conflict in rhesus groups, as shown by an inverse relationship between rate of successful interventions and group-level conflict severity and wounding. However, intervention type was not distinguished in this study, so the relative contribution of impartial intervention to group stability has not been evaluated [21].

Let’s consider the costs and benefits of impartial interventions in rhesus macaques. The *group stability hypothesis* predicts the only benefit to the intervener is the group-level benefit of reduced conflict as well as low cost of performance. In contrast, potential ‘selfish’ benefits of impartial interventions include dominance or mating benefits. First, ‘policing’ may reinforce one’s dominance by interfering with others’ efforts to rise up the social hierarchy, in fallow deer, *Dama dama* [22]. This *dominance assurance hypothesis* predicts selective targeting of direct social competitors, such as male rhesus selectively intervening in male-male fights and female rhesus selectively intervening in female-female fights. Second, ‘policers’ may improve their access to mates if impartial intervention increases the chance of mating with the participants. This *mating benefits hypothesis* predicts preferential intervention in fights with opposite sex participants, and an increased chance of mating with that participant(s) relative to those who do not impartially intervene. Lack of both selective targeting and increased access to mates would support the *group stability hypothesis* [12], [16]. See Table 1.

**Table 1:** Proposed hypotheses regarding the function of impartial interventions in rhesus macaques.

Hypothesis	Benefit to intervener	Benefit to group	Dyads policed
group stability	reduced fighting & trauma	reduced fighting & trauma	all sex-dyads targeted equally
dominance assurance	reinforce individual rank	no benefit	males: mm females: ff
mating benefits	increased chance of mating	no benefit	males: ff or mf; females: mm or mf

#### Partial intervention

Partial interventions involve an intervener supporting either the dominant participant or the subordinate participant. As stated above, partial interventions (often referred to as agonistic support) have not been previously considered as policing because choosing sides is assumed to confer additional (i.e. selfish) benefits which serve as the primary selective force on the behavior. However, certain types of partial interventions have significant potential to be prosocial. In fact, support of subordinates or victims has previously been thought to serve a conflict control function [15], [23], [24], perhaps because there appears to be little chance to gain dominance benefits or inclusive fitness benefits via kin selection. We propose a *policing support hypothesis* that includes these types of partial interventions as policing.

Two types of support have the potential to function as policing: (1) support of non-kin subordinates in polyadic fights (hereafter SNP support) and (2) support of non-kin subordinates in dyadic fights (hereafter SND support). First, support of non-kin eliminates the potential to benefit via kin selection. Second, some fights are more costly to the group than others. Polyadic fights trigger increased redirection and contact aggression among group members, which appears to underlie cascades of aggression in at least pigtail macaques [25]. Indeed, polyadic fights are policed more than dyadic fights in chimpanzees [12]. However, most fights do not require policing, as natural conflict resolution is important for maintenance of relationships [26]. Support in polyadic fights may serve a policing function, but this likely depends upon who is supported. Support of the dominant likely reinforces the hierarchy and/or the intervener’s rank, whereas support of the subordinate has less obvious additional benefit. Finally, SND support may be policing because some dyadic fights might be as harmful to group stability as polyadic fights. Under the *policing support hypothesis*, we predict SNP support and SND support (a) are low cost, at least for highest-ranking interveners, (b) are positively associated with lower rates of group-level conflict, and (c) show no preferential targeting.

Let’s consider alternative benefits that interveners may receive by providing SND or SNP support. First, supporting a subordinate might confer dominance benefits. Let’s consider monkeys A, B and C, such that A>B>C. In one scenario, B could support C against A and benefit by challenging A’s position and eventually rise in rank. However, policers typically outrank both conflict participants because only the highest-ranking group members have the power to stop others’ fights [8], [12]. Therefore, such scenarios are unlikely to constitute policing and will not be considered further here. In a second scenario, A supports C against B to reinforce his dominance over B. In this scenario, the only plausible reason for A to reinforce his dominance over B by involving C would be if A’s dominance over B is somewhat ambiguous. If A’s dominance over B is settled, then getting involved in C’s conflict with B entails unnecessary risk. Under this *dominance ambiguity reduction hypothesis* that interveners support subordinates to reduce the degree of dominance ambiguity with the target, we predict that most SND and SNP support involves an intervener and target with a high degree of dominance uncertainty (measured via dominance transitivity pathways; [27]).

Second, under the *mating benefit hypothesis* we predict that (a) most SND and SNP support occurs during the mating season, (b) interveners support opposite-sex individuals, and (c) a subordinate that receives support during the mating season is more likely to mate with his/her supporter than if no support was given. A final hypothesis is that interveners support subordinates with whom they have a strong social bond, e.g. affiliative partners. Under this *social bond hypothesis*, we predict that support of subordinate non-kin occurs more frequently between interveners and subordinates who groom frequently. See Table 2.

**Table 2:** Proposed hypotheses regarding the function of support of subordinate non-kin in rhesus macaques.

Hypothesis	Benefit to intervener	Benefit to group	Who is supported?
policing support	reduced fighting & trauma	reduced fighting & trauma	no preferential support
dominance ambiguity	reinforce individual rank	no benefit	target whose subordinance is ambiguous
mating benefits	increased chance of mating	no benefit	support opposite sex
social bond	maintain important social bond	no benefit	support frequent grooming partner

An alternative scenario is that conflict management is not accomplished by policing, but by maintenance of the dominance hierarchy. Flack and de Waal [20] predict the variance in social power in rhesus macaques is too uniform to permit low-cost impartial policing by high-ranking individuals. Instead, conflict control in rhesus macaques is predicted to occur by reinforcement of dominance relationships [20]. As such, intervention behavior may still be associated with group stability, but for different reasons. Supporting a dominant conflict participant or kin reinforces existing ranks as well as the matrilineal structure of the hierarchy [28] and can prevent lower-ranking individuals from advancing in rank [22], [29]. Under this alternative *dominance maintenance hypothesis*, we predict groups have lower rates of severe aggression, wounding, and social relocation if they have higher rates of support of kin or dominants in dyadic fights.

Below, we test whether impartial interventions, SND and SNP support are prosocial policing by analyzing intervention types in relationship to: (1) measures of group-level stability, e.g. severe aggression and wounding, (2) sex-dyad combinations targeted by interveners, (3) dominance ambiguity between intervener and target, (4) mating between intervener and beneficiary, and (5) policing cost.

## Methods

### Ethics Statement

All research reported here adhered to the recommendations in the *Guide for the Care and Use of Laboratory Animals of the National Institutes of Health,* the laws of the United States government, and the recommendations of the Weatherall report, “The use of non-human primates in research”. All subjects were housed in large social groups in half-acre outdoor enclosures with natural substrate to provide for their psychological well-being. Each outdoor enclosure included ten A-frame houses, multiple suspended barrels and swings, and several perches. Monkey chow was provided twice daily, at 0700 h and between 1430 and 1530 h. Additional food enrichment (fresh fruit, vegetables, or seed mixture) was provided daily. Water was available ad libitum via six widely-spaced water spigots. This study was purely observational; it involved no experimental or invasive treatment or sacrifice of the animals. All occurrences of illness or injury among study subjects were immediately reported to and treated by CNPRC veterinary staff, and all efforts were made to ameliorate suffering. This project was approved by the University of California, Davis Institutional Animal Care and Use Committee, protocol #11843.

### Data Collection

Subjects were seven social groups of rhesus macaques (108–197 individuals) at the California National Primate Research Center studied between June 2008 and December 2009 for a total of 1500 hours (Table 3). An event sampling design was used to record aggressive and submissive interactions. Aggression included threat, threat and follow, lunge, chase <3 meters, chase >3 meters or grapple, bite <5 seconds, chase and bite <5 seconds, and bite >5 seconds. Submission included silent bared teeth display (SBT), turn away, turn away with SBT, move out of arms’ reach, move out of arms’ reach with SBT, run away <3 meters, run away <3 meters with SBT, run away >3 meters, run away >3 meters with SBT, prolonged scream, crouch (animal stops resisting aggression, i.e. mobbing events), and crouch with SBT. Severe aggression included any interaction involving a bite. Each group was observed for six hours on four days per week for one week of each month during each group’s study period.

**Table 3:** Group-level characteristics.

Group	Mean Group Size	Severe Aggression count	Trauma count	Social relocation count
1B	177.6	403	37	2
5	136.6	331	54	5
8	156.9	445	27	2
10B	164.9	605	110	8
14B	108.3	306	10	1
16D	149.4	344	54	8
18B	197.2	395	42	6

Conflict events were recorded as a series of pairwise agonistic interactions linked by both temporal proximity (within 30 s) and common participants (A threatens B, 20 s later A threatens C were considered the same conflict event). Each conflict event could be composed of one or more dyads. A total of 17,989 conflict events were recorded across the seven study groups, 10,247 of which involved a single dyad (57.0%) and 7,742 of which involved more than one dyad (43.0%). Intervention was defined as a third-party entering an on-going fight by directing aggression at, directing submission at, affiliating with (groom, social contact, present rump), or approaching one or both of the combatants. A total of 5,485 interventions were recorded, including 440 impartial and 5,045 partial interventions (see Table 4). The total number of participants, i.e. the fight size, ranged from 2 to 15 with a mean of 3.8 participants per fight. Successful interventions stopped the targeted fight within 5 seconds of intervening, whereas the targeted opponents continued fighting in failed interventions. For fights with more than two participants, successful interventions were those in which the targeted set of participants stopped fighting, regardless of whether non-targeted participants continued to fight.

**Table 4:** Counts of the frequency of each intervention type across the seven study groups.

	1B	5	8	10B	14B	16D	18B
Impartial	74	54	74	56	90	25	67
Subordinate non-kin dyadic	37	49	48	42	36	12	47
Subordinate non-kin polyadic	99	79	91	56	81	31	73
Dominant non-kindyadic	102	87	77	49	74	35	71
Dominant non-kin polyadic	156	173	167	60	166	81	164
Subordinate kin dyadic	95	71	118	57	114	27	74
Subordinate kinpolyadic	87	100	118	62	97	40	109
Dominant kin dyadic	66	48	50	70	92	30	64
Dominant kin polyadic	109	108	116	85	159	86	110

### Individual-level Behavioral Variables

#### Rank

Ranks were determined using dyadic aggressive interactions with a decisive outcome using a social network approach which incorporates information from indirect transitivity pathways into the standard win/loss matrix [27]. The highest rank = 1.

#### Dominance ambiguity

The probability that one animal is dominant over another was calculated for all dyads using dominance transitivity from the aggression network, whereby multiple indirect dominance pathways (via common third parties) were used to infer missing data in the win/loss matrix [27]. Dominance probabilities range from 0 to 1, where 0 indicates that *i* is completely submissive to *j*, 1 indicates that *i* is completely dominant to *j*, and 0.5 indicates complete dominance ambiguity.

#### Mating frequency

A binary variable of whether a dyad was observed mating or consorting. Dyads were scored as having mated if (1) sexual mounting occurred or (2) the dyad showed evidence of a consortship, including constant mutual maintenance of proximity during the mating season, coordinated movement and foraging behaviors, and frequent grooming.

#### Intervention cost

A measure of the average severity of return aggression received by each intervener from their targets. Aggression severity ranged from 0–8 (see above), and average severity per intervener was calculated for each intervention type.

#### Intervention type

Categorical variables describing the type of behavior shown by the intervener with respect to four factors: partiality, kinship, dominance, and fight size.

- Partiality: support interventions were those in which the intervener sided with one of the conflict opponents whereas impartial interventions were those in which the intervener showed no partial treatment. Impartial interventions could be passive (approaching the fight) or aggressive (directing aggression at both participants).
- Kinship: Two individuals were defined as kin if they were from the same matriline. Average matriline relatedness ranged from 0.5 (mother-daughter pairs) to 0.08, matrilines with multiple branches in which the matriarch was absent. Males in these captive groups cannot disperse, therefore both females and natal males could have maternal kin present in the group. Each group also included 1–5 unrelated adult males.
- Dominance: whether the intervener supported the subordinate or dominant conflict opponent.
- Fight size: The number of conflict opponents in the targeted fight: dyadic (2) or polyadic (3+). A threatens B and B redirects to C is polyadic, as is A threatening both B and C.

### Group-level Behavioral Variables

#### Intervention type success

The rate of success for each intervention type (see above) across the study period was calculated for all seven groups.

#### Severe aggression

The rate of severe aggression (bite, attack or long chase) per individual per hour across the study period was calculated for all seven groups. Daily tallies of average group size were used for all calculations of group-level rates.

#### Wounding

The rate of wounding (physical injury requiring hospitalization, e.g. laceration) per individual across the study period was calculated for all seven groups. Wounding rates were calculated from CNPRC hospital records.

#### Social relocation

The rate of social relocation (permanent removal of individuals from the group for social reasons) per individual across the study period. Such removals were animals that were either frequent targets of aggression or frequent instigators of aggression such that removal was deemed beneficial for the health/well-being of both the individual and the group. Decisions for removal were made by CNPRC veterinarians and behavioral management staff.

### Analyses

For group-level analyses, we fit linear regression models to each of the three stability measures: severe aggression rate, wounding rate, and social relocation rate. We fit a maximum of two variables in each model due to the small sample size of seven groups, which precluded standard interaction terms among intervention type categories. We instead calculated the rate of each two-way and three-way combination of intervention type categories. For example, we calculated the rate of successful SNP support (three-way combination) as well as the rate of successful support of non-kin in polyadic fights regardless of dominance (two-way combination). AIC scores were used to select the best-fit model [30].

For analyses of preferential targeting, we counted the number of times males and females each targeted male-male, male-female, and female-female dyads using impartial interventions (to test *group stability* vs. *dominance assurance* vs. *mating benefit* hypotheses), as well as those providing SND and SNP support (to test the *policing support hypothesis*). We used Chi-square tests to determine whether each intervention type targeted each sex-dyad more or less often than expected, given their overall frequency. Since conflict events were recorded as a series of pairwise interactions, the overall frequencies were calculated as the number of male-male, male-female, and female-female pairwise interactions across all dyadic and polyadic events.

For analyses of dominance ambiguity, mating benefit, strong social bond, and intervention cost, we fit multi-level Poisson or logistic regression models [31] using intervener, beneficiary or target (as appropriate), and group ID as random effects. Dependent variables were: the frequency of targeting the dominant conflict participant across all possible dyads, whether or not each male-female dyad mated (yes/no), the frequency of SND or SNP support across all dyads, and the average severity of return aggression received per intervener. Fixed effects varied by analysis but generally included attributes of the intervener, beneficiary and/or target (sex, rank, age), attributes of the dyad (dominance ambiguity, groom frequency, interaction frequency, frequency of support during the mating season and outside of the mating season), and attributes of the intervention (type, severity of aggression by intervener, total intervention frequency for each intervener across each intervention type), as well as interactions among these main effects. We used AIC scores to select the best fit model, i.e., the model with the lowest AIC score [30].

## Results

### Group Stability Measures

To test the *policing support hypothesis*, we fit a linear regression model to the group-level rate of severe aggression and report the results of the best fit model (AIC = 3.4, compared to the second best fit model ΔAIC = 5.7; N = 7 groups). Groups with a higher rate of successful support to non-kin in polyadic fights had lower rates of severe aggression (β = −657.1, p = 0.005), whereas groups with a higher rate of successful support to kin in dyadic fights had higher rates of severe aggression (β = 1015.1, p = 0.007). This means that a group with the maximum observed rate of 0.005 successful support of non-kin in polyadic fights per individual per hour will have 2.75 times less severe aggression per individual per hour than a group with the minimum observed rate of 0.001 (1.50 vs. 4.11 severe aggression per ID per hr). The top five best fit models are presented in Table S1.

To further test the *policing support hypothesis*, we fit a linear regression model to the group-level rate of wounding and report the results of the best fit model (AIC = −18.6, compared to the second best fit model ΔAIC = 5.9; N = 7 groups). Groups with a higher rate of successful impartial interventions in polyadic fights had a lower rate of wounding (β = −859.0, p = 0.0008), whereas groups with a higher rate of successful support of dominant kin had higher rates of wounding (β = 314.6, p = 0.003). This means that a group with the maximum observed rate of 0.0009 successful impartial interventions in polyadic fights per individual per hour has 45 times less wounding per individual than a group with the minimum observed rate of 0.0004 (0.02 vs. 0.95 trauma per individual). The top five best fit models are presented in Table S2.

Finally, we fit a linear regression model of the group-level rate of social relocations and report the results of the best fit model (AIC = −50.6, compared to the second best fit model ΔAIC = 5.5). Groups with a higher rate of successful support to non-kin subordinates had a lower rate of social relocations (β = −49.9, p = 0.001), whereas groups with a higher rate of successful support of dominants in dyadic fights had higher rates of social relocations (β = 41.4, p = 0.005). This means a group with the maximum observed rate of 0.003 successful support of subordinate non-kin per individual per hour has 13.5 times fewer social relocations than a group with the minimum observed rate of 0.001 (0.108 vs. 0.008 social relocations per individual). The top five best fit models are presented in Table S3.

### Intervener Sex and Sex-dyad Combinations

Across the seven study groups, we recorded 219 impartial interventions targeting fights involving non-kin (169 polyadic, 50 dyadic). Males performed 58.4% of these impartial interventions (Table 5), and all sex-dyad combinations were targeted. Male interveners targeted female-female dyads more often than expected and male-female dyads less often (chi-square = 5.89, df = 2, p = 0.05), whereas female interveners targeted each sex-dyad as expected (chi-square = 3.08, df = 2, p = 0.21).

**Table 5:** Observed frequency of targeting each sex-dyad combination across intervention types.

	Impartial non-kin		SNP support		SND support	
Sex-dyad	Male^ab^	Female	Male	Female	Male	Female
mm	12 (14.8)	15 (11.5)	40 (34.1)	**49 (34.8)**	17 (15.0)	12 (11.3)
mf	**36 (46.9)**	25 (32.4)	100 (98.8)	92 (95.0)	43 (55.4)	51 (42.8)
ff	**80 (66.3)**	51 (47.1)	120 (127.1)	109 (120.2)	94 (83.6)	54 (62.9)
Total	128	91	260	250	154	117

a Values highlighted in **bold** differed significantly from expected in Chi-square tests.

b Expected values are given in parentheses.

Since the policing support hypothesis regarding partial interventions also predicts a lack of preferential targeting, we analyzed expected versus observed frequencies of SND and SNP support. We recorded 510 instances of SNP support, and males performed 51.0%. Male interveners targeted each sex-dyad as expected (chi-square = 1.43, df = 2, p = 0.49), whereas female interveners targeted male-male fights more often than expected (chi-square = 8.16, df = 2, p = 0.02).

We recorded 271 instances of SND support, 56.8% performed by males. Chi-squared tests showed that both male and female interveners targeted each sex-dyad as frequently as expected (males: chi-square = 4.3, df = 2, p = 0.11; females: chi-square = 3.6, df = 2, p = 0.16).

### Dominance Ambiguity Reduction

Most SND and SNP support did not target individuals whose dominance status was ambiguous relative to the intervener. Of the 510 instances SNP support and 271 instances of SND support, 44 SNP and 20 SND involved an intervener and target with ambiguous dominance (i.e. 0.4< d <0.6) and 56 SNP and 24 SND involved a target that outranked the intervener (i.e. d <0.4). The remaining 410 instances of SNP support and 227 instances of SND support were given by interveners that unambiguously outranked the target.

We fit a multi-level Poisson regression model to the dyadic frequency of SND and SNP support to evaluate whether interveners preferentially support subordinates whose opponents had an ambiguous dominance relationship with the intervener (N = 16,274 intervener-target dyads). Dyadic and polyadic fights were analyzed separately. The best fit model (AIC = 2083, compared to second best fit model ΔAIC = 3) of SNP support showed that interveners were more likely to intervene against targets with whom their dominance probability was higher (d: β = 2.75, p<0.001). A significant interaction dominance probability×frequency peaceful submission indicated that among dyads with a high frequency of peaceful submission from target to intervener (>6 submissions), dominance probability did not influence likelihood of intervening against the target (peaceful submission: β = 0.42, p = 0.01; d×peaceful submission: β = −0.41, p = 0.02). See Table S4 for detailed model output and Table S5 for the top five best fit models.

The best fit model (AIC = 1357, compared to second best fit model ΔAIC = 2) of SND support showed the same pattern as for polyadic fights: interveners were more likely to intervene against targets with whom their dominance probability was higher (d: β = 2.09, p = 0.009). A significant interaction dominance probability×frequency peaceful submission indicated that among dyads with a high frequency of peaceful submission from target to intervener (>6 submissions), dominance probability did not influence likelihood of intervening against the target (peaceful submission: β = 0.50, p = 0.03; d×peaceful submission: β = −0.48, p = 0.05). See Table S6 for detailed model output and Table S7 for the top five best fit models.

### Mating Benefit

SND and SNP support was distributed across all seasons such that 44.3% of SND support and 45.3% of SNP support occurred during the mating season (September – November). Male and female interveners both supported to male and female beneficiaries. In dyadic fights, both male and female interveners supported each sex at similar rates (male interveners: 19.0% support male; female interveners 23% support male). The same was true for polyadic fights (male interveners: 32.0% support male; female interveners: 35.6% support male).

**Figure 1:**
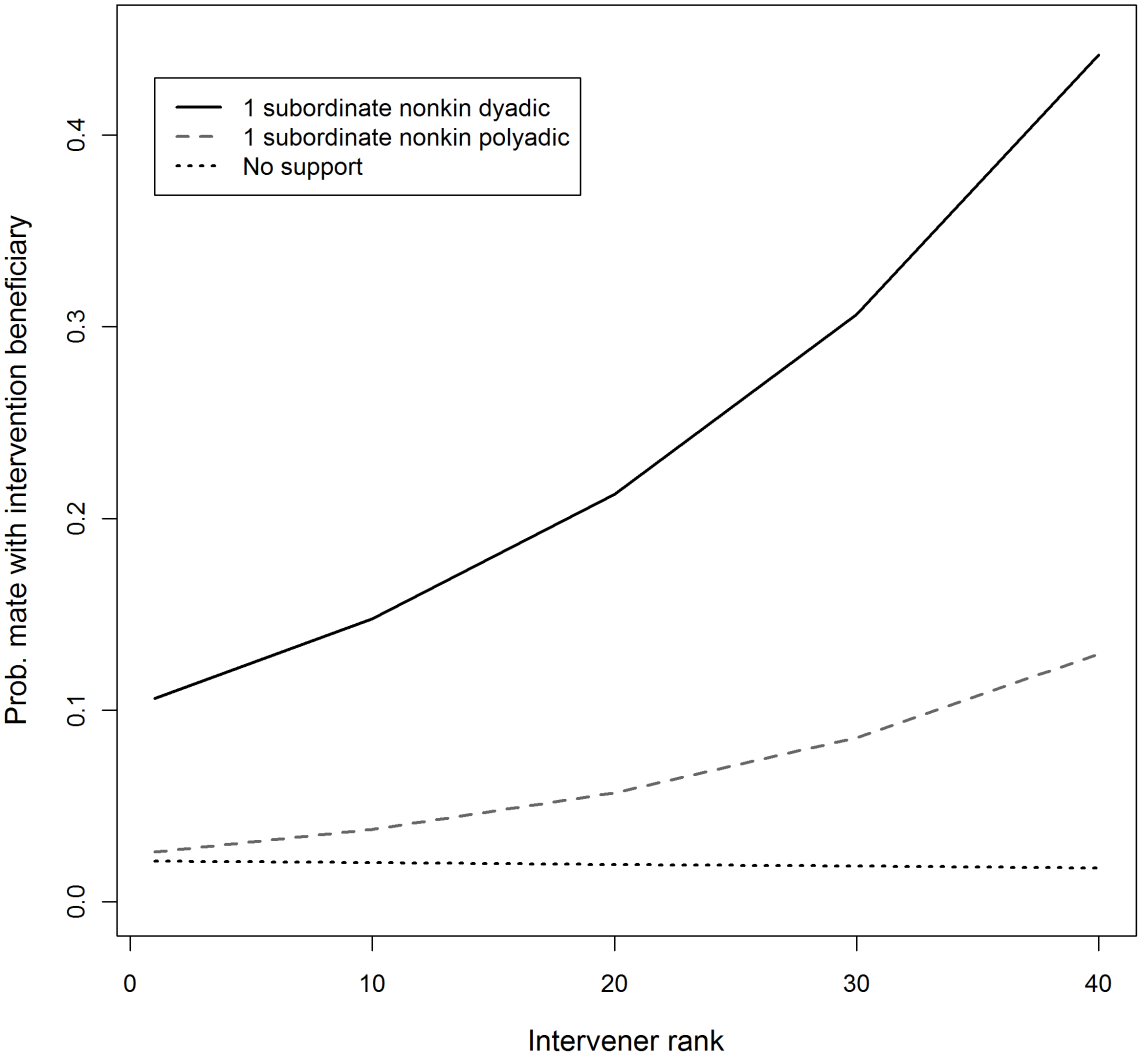
Expected mating between intervener and beneficiary (from model coefficients) plotted by intervener rank for several intervention types.

We fit a multi-level logistic regression model to whether male-female dyads were observed to mate (yes/no) to test the prediction that interveners support opposite-sex subordinate to increase their chances of mating with that individual (N = 5309 male-female dyads). The best fit model (AIC = 1730, ΔAIC = 3) showed that high-ranking interveners that provided opposite-sex SNP support were not more likely to mate with the beneficiary than individuals that never provided such support (β = 0.17, p = 0.8), but SNP support did improve low-ranking interveners chances of mating with the beneficiary (rank×SNP: β = 0.045, p = 0.04; Figure 1). For SND support, all interveners were more likely to mate with the beneficiary if support was provided during the mating season, but this effect was greater among lower-ranking interveners (SND: β = 1.57, p = 0.01; rank×SND: β = 0.04, p = 0.1). Although impartial intervention frequency during the mating season was part of the model, it was not significant (β = 0.73, p = 0.2). Notably, frequencies of support provided outside the mating season for dyadic and polyadic fights were not significant and not part of the best-fit model. See Table S8 for detailed model output and Table S9 for the top five best fit models.

### Strong Social Bond Benefit

Overall, most SND and SNP support occurred in dyads with no affiliative relationship, and thus likely no strong social bond. Of the 510 instances of SNP support, 70.4% (N = 359) of the intervener-beneficiary pairs had never been observed to groom and 66.4% (N = 339) had never been observed in any affiliative contact. Of the 271 instances of SND support, 68.6% (N = 186) of the intervener-beneficiary pairs had never been observed to groom and 65.7% (N = 178) had never been observed in any affiliative contact.

**Figure 2:**
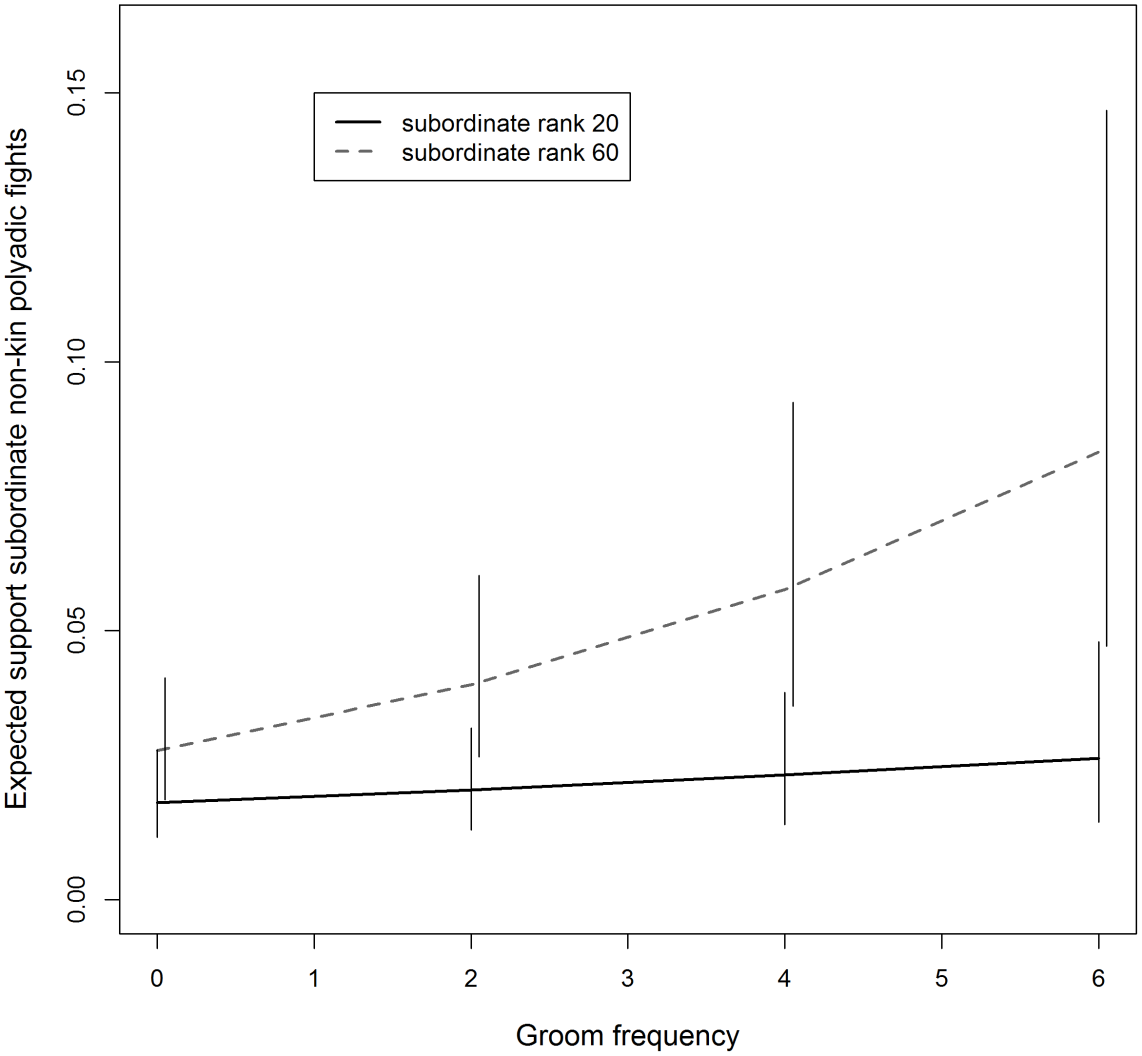
Expected frequency of SNP support plotted by intervener-beneficiary groom frequency for beneficiaries of high and low rank.

We fit multilevel Poisson regression models to the dyadic frequencies of SND and SNP support to evaluate whether interveners are more likely to support subordinates with whom they groom frequently. SND and SNP support were analyzed separately. The best fit model (AIC = 2147, compared to second best fit model ΔAIC = 3) of SNP support included an interaction term beneficiary rank×total groom which showed that only among low-ranking beneficiaries was SNP support more likely in dyads that groom frequently (>5 groom events) (total groom: β = 0.003, p = 0.90; benf. rank: β = 0.011, p = 0.008; total groom×benf. rank: β = 0.003, p = 0.01; Figure 2). See Table S10 for detailed model output and Table S11 for the top five best fit models.

**Figure 3:**
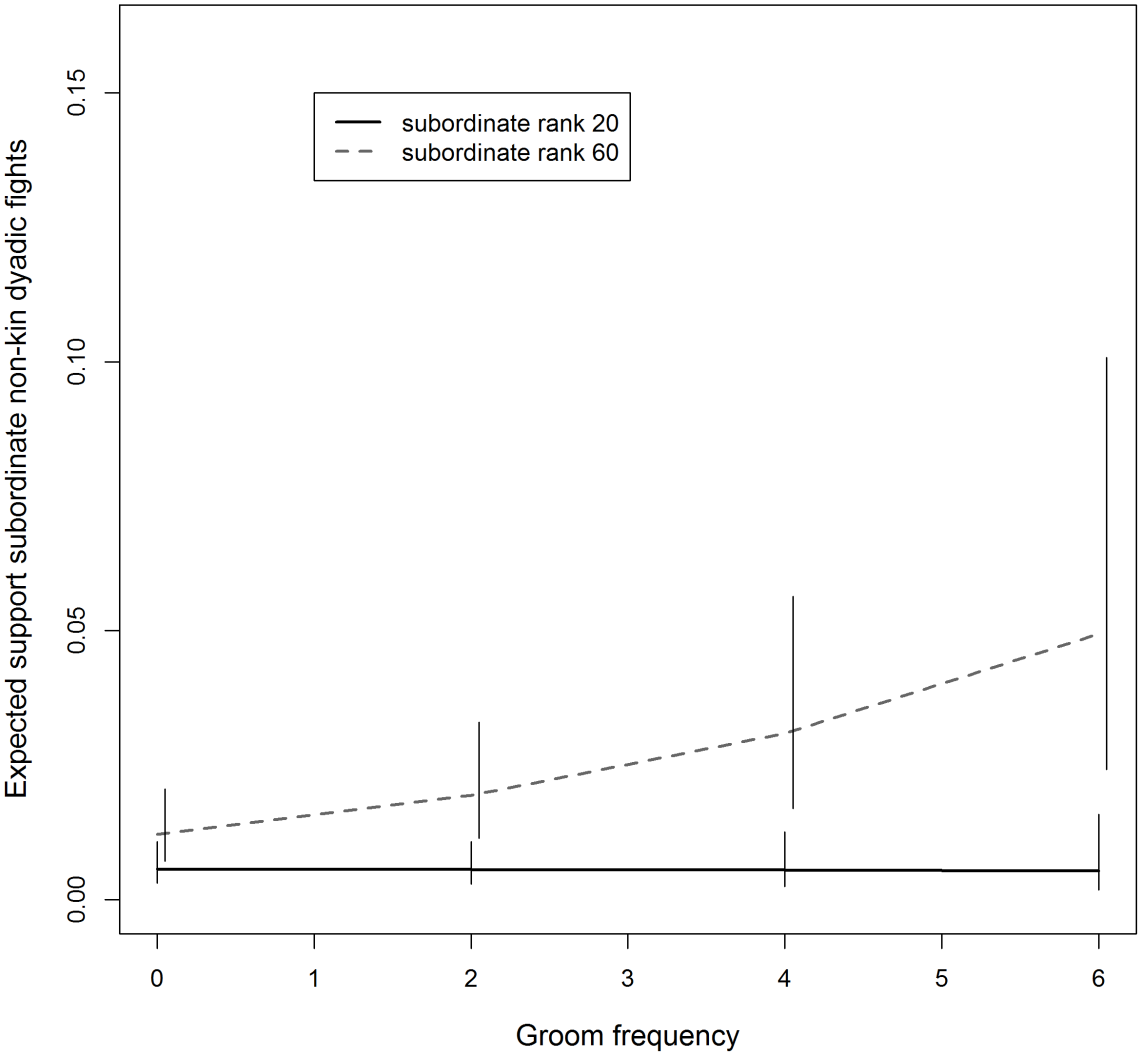
Expected frequency of SND support plotted by intervener-beneficiary groom frequency for beneficiaries of high and low rank.

The best fit model of SND support showed the same pattern as for polyadic fights (AIC = 1178, compared to second best fit model ΔAIC = 11). The interaction beneficiary rank×total groom showed that only among low-ranking beneficiaries was SND support more likely in dyads that groom frequently (total groom: β = −0.13, p = 0.29; benf. rank: β = 0.019, p = 0.003; total groom×benf. rank: β = 0.006, p = 0.004; Figure 3). Intervener-beneficiary pairs with more than 5 grooming events were rare: 10 instances of support in polyadic fights and 9 instances of support in dyadic fights, further showing that SND and SNP support are likely to be policing because such support is rarely given to strong social affiliates. See Table S12 for detailed model output and Table S13 for the top five best fit models.

We took the full data set of 510 instances of SNP support and 271 instances of SND support and categorized each intervention by whether additional benefits could be gained (i.e. dominance ambiguity reduction, mating, or strong social bond). A total of 408 of the 510 instances of SNP support and 167 of the 271 instances of SND support had no additional benefits that could be gained by the intervener and involved an intervener that outranked the target. For impartial interventions, the intervener outranked both conflict participants in 194 of the 219 interventions involving non-kin. Therefore, we recorded a total of 769 prosocial policing interventions (408 SNP +167 SND +194 impartial), which represents 14.0% of all observed interventions (N = 5,485 interventions).

### Policing Cost

Overall, impartial interventions and SND and SNP support were less costly than support of kin subordinates. Interveners received return aggression from targets in 3 of the 440 impartial interventions (0.7%), 22 of the 298 support of non-kin subordinates in dyadic fights (7.4%), 44 of the 510 SNP support (8.6%), and 253 of the 556 SND support (45.5%).

**Figure 4:**
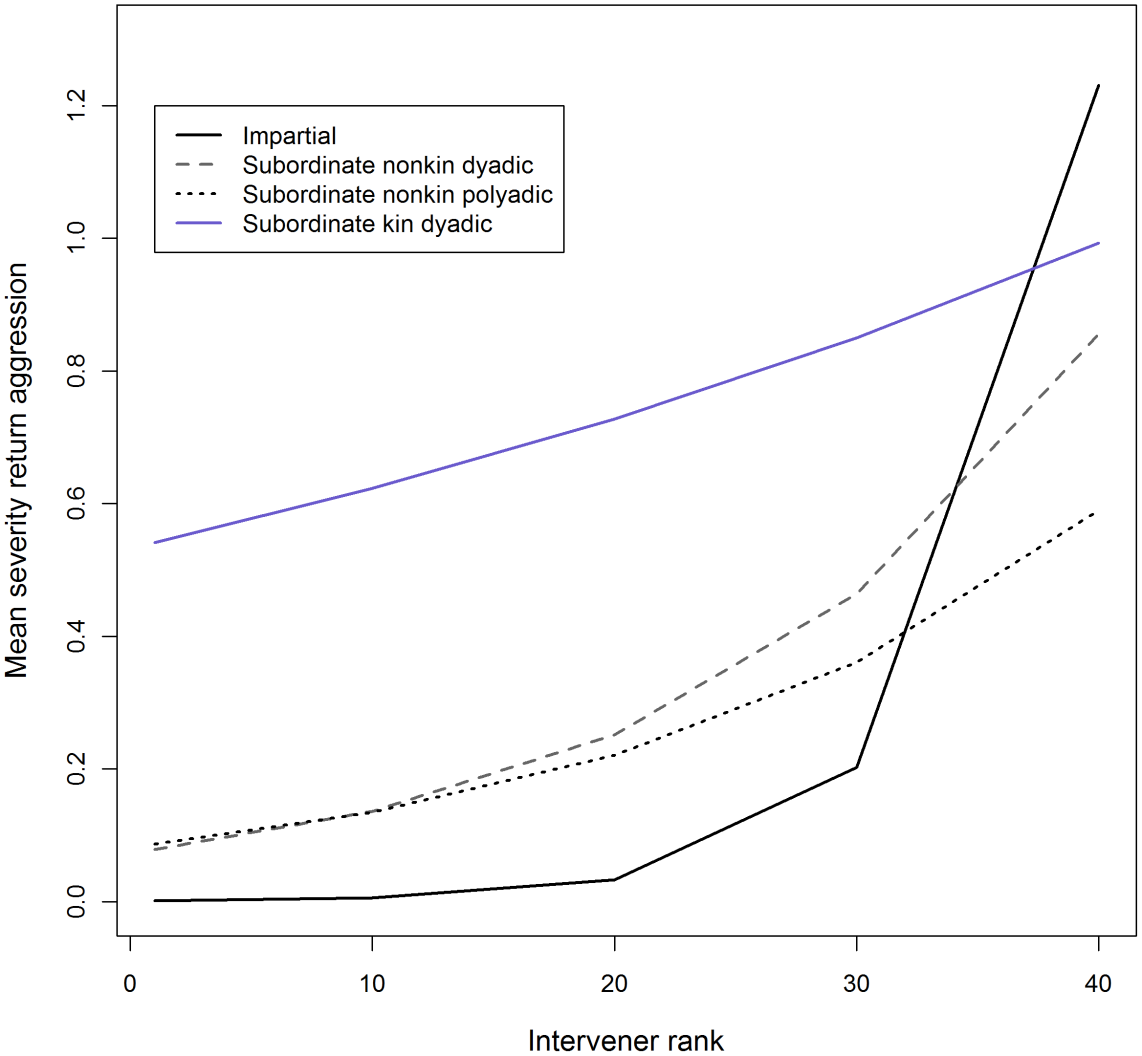
Expected intervention cost (from model coefficients) plotted by intervener rank for several intervention types.

We fit a multilevel Poisson regression model to the average severity of return aggression across four intervention types to see if policing is low-cost for the highest-ranking interveners (N = 832 interveners with at least 1 intervention). The best fit model (AIC = 880.8, compared to the second best fit model ΔAIC = 3.9) included the three-way interaction term intervener rank×intervention type×intervention frequency which showed that among high-ranking individuals, support of kin in dyadic fights was more costly than impartial interventions (impartial vs. subordinate kin dyadic: β = −7.2, p<0.0001), SND and SNP support (subordinate kin dyadic vs. SNP: β = −1.6, p<0.0001; subordinate kin dyadic vs. SND: β = −1.9, p<0.0001; Figure 4). The cost changes for each intervention type among lower-ranking animals such that cost initially converges for all intervention types near rank 40 (Figure 4) and among the lowest-ranking individuals support of subordinate kin is less costly than impartial and policing support interventions. Among impartial interventions, higher intervention frequency was associated with greater cost, and this was most pronounced among low-ranking animals (frequency: β = 0.26, p = 0.01; rank×frequency: β = 0.037, p = 0.01; rank×frequency×impartial: β = 0.037, p = 0.02; rank×frequency×SND: β = 0.01, p = 0.12; rank×frequency×SNP: β = 0.006, p = 0.07). Frequency of support was not significant for support of subordinate kin in dyadic fights or subordinate non-kin in both dyadic and polyadic fights (frequency [subordinate kin]: β = 0.10, p = 0.3; frequency [SND]: β = 0.02, p = 0.7; frequency [SNP]: β = 0.11, p = 0.3). See Table S14 for detailed model output and Table S15 for the top five best fit models.

## Discussion

We investigated intervention behavior in seven captive groups of rhesus macaques to evaluate whether impartial interventions, SND support and SNP support serve the primary social function of maintaining group stability, as has been shown for impartial interventions in some other species. Overall, our analyses support the *group stability* and *policing support hypotheses*, impartial interventions and SNP and SND support serve to manage group-level conflict. Group-level analyses tie these types of intervention to lower levels of conflict. Dyadic-level analyses show that neither impartial interventions nor SND and SNP support confer additional benefits, beyond the group-level benefit, to high-ranking interveners. And only in certain circumstances to SND and SNP support confer additional benefits to low-ranking interveners. The group-level and dyadic-level analyses, in conjunction with the cost analyses, all point toward impartial interventions and SND and SNP support serving a policing function (see Table 6).

**Table 6:** Summary of results across intervention types.

	Impartial	SNP support	SND support
Group-level benefit?	Fewer traumas	Less severe aggression	Fewer relocations
Preferential targeting?	Males: target mf dyads; Females: no preference	Males: no preference; Females: target mm dyads	Males: no preference; Females: no preference
Target ambiguous?	–	No	No
Mating benefit?	No	Yes: low-rank in mating season	Yes: in mating season
Help social bond?	–	Yes: low-rank beneficiaries only	Yes: low-rank beneficiaries only
Cost	Low-cost for high rankers	Low-cost for high rankers	Low-cost for high rankers
Prosocial?	Yes	Yes, except by low-rank in mating season	Yes, except in mating season

### Group-level Stability Measures

Our group-level analyses of trauma, severe aggression, and social relocation verify the *group stability* and *policing support hypotheses* that impartial interventions and support of subordinate non-kin, respectively, serve to manage group conflict. Groups with higher rates of impartial interventions in polyadic fights had lower rates of trauma, consistent with previous findings that impartial interventions are associated with reduced severity and frequency of aggression [12], [16]. Polyadic fights trigger increased redirection and contact aggression among group members [25]; thus impartial interventions that stop polyadic fights are associated with lower rates of trauma. However, higher rates of supporting dominant kin were associated with higher rates of trauma, indicating that interventions which help to reinforce the hierarchy (at least the matrilineal structure of the hierarchy) do not contribute to conflict management as originally predicted by Flack and de Waal [20].

SNP support best predicted severe aggression at the group level – groups with higher rates of SNP support had lower rates of severe aggression. As mentioned above, polyadic fights are more costly to the group and thus their termination has an obvious link to lower rates of severe aggression. Less obvious is why SNP support better suppresses severe aggression than impartial interventions. Severe aggression includes long chasing, attacking and biting, not all of which cause injury. Thus, while impartial interventions on polyadic fights only reduce severe aggression leading to trauma, support of non-kin in polyadic fights reduces all types of severe aggression. Severe aggression was also positively associated with the rate of supporting kin in dyadic fights. In 62% of these interventions, interveners supported a kin subordinate who initiated a fight against a dominant animal, which likely has the effect of exacerbating serious aggression as the fight counters the established hierarchy. Subordinate kin support may underlie this positive association of kin support with severe aggression at the group level.

The rate of supporting non-kin subordinates, regardless of fight size, was the best predictor of social relocations – groups with higher rates of supporting non-kin subordinates had *lower* rates of permanent relocation of animals from the group. This association is likely due to the fact that animals are selected for social relocation if they are (a) frequent targets of aggression, or (b) frequent instigators of serious aggression. Interveners that support non-kin subordinates may be preventing individuals from becoming ‘instigators’ or ‘targets’. Additionally, the rate of supporting dominants in dyadic fights was positively associated with social relocations. In 86% of these interventions, interveners supported an initiator who was at least 10 ranks higher than the recipient, indicating that (a) this support merely reinforced well-established dominance ranks, and (b) beneficiaries did not appear to require coalitionary support to win the fight. The positive association between support of dominants and social relocation may be due to interveners extending the duration of aggression in the fight beyond the initiator’s duration of aggression.

### Absence of Dominance Benefits

The analyses of preferential targeting and the analyses of intervener-target dominance ambiguity showed a lack of dominance benefits to policers via impartial intervention or SNP and SND support, respectively. This is consistent with findings in chimpanzees that impartial interventions target all sex dyads [12]. First, although impartial interventions by males differed from expected values, this was due to males targeting female-female dyads more often than expected, rather than male-male dyads as predicted by the *dominance assurance hypothesis*. Furthermore, although preferential targeting of female-female dyads is consistent with the *mating benefits hypothesis*, analysis of actual mating behavior showed that impartial interventions did not increase a male intervener’s chances of mating with those females (see below). SND and SNP support also showed no preferential targeting, consistent with the *policing support hypothesis*. Second, SND and SNP support was not associated with targeting those whose dominance relationship with the intervener was ambiguous, meaning that interveners were not selectively offering support to the beneficiary in order to reinforce their dominance over the target. The majority of SND and SNP support (SND: 83.8%, SNP: 80.4%) was performed by interveners that unambiguously outranked the target, which suggests that the intervener gained very little in terms of dominance reinforcement. In fact, interveners tended to support subordinates when their target was clearly subordinate to the intervener, suggesting that interveners may selectively police fights they stand a good chance of winning. It is unlikely that the reason for targeting a clearly subordinate individual is to reinforce dominance rank. Such a choice adds an element of complexity and risk that is unnecessary, especially when the intervener could simply initiate a direct fight with the target at some other point. There were a small percentage of instances where the intervener-target relationship was ambiguous, and these are likely not prosocial in nature and, therefore, not policing.

### Limited Mating Benefit

Mating analyses showed that impartial interventions and most SND and SNP support did not confer mating benefits to the intervener. Increased chance of mating with the beneficiary appears to occur under limited circumstances. Specifically, all SND support provided during the mating season was associated with increased chance of mating with the beneficiary, but support provided during other seasons had no such benefits. In addition, SNP support by low-ranking interveners also increased their chances of mating with the beneficiary, but like SND support, this was only true during the mating season. This mating benefit, however, applies to only a portion of subordinate non-kin support –28.0% of SND support was given to opposite-sex beneficiaries during the mating season and the remaining 72% represents prosocial policing support. Similarly, 22.4% of SNP support was given during the mating season. Furthermore, half of that 22.4% (57 of 114) was given by the top-10 highest-ranked interveners, indicating that only 11.2% of SNP support conferred a mating benefit to the intervener, while the remaining 88.8% constituted prosocial policing support.

### Little Support of Strong Social Affiliates

Some intervention support was given to beneficiaries that had a strong social relationship with the intervener, suggesting that these instances of support were not prosocial in nature. However, like all of the previously described analyses of SND and SNP support, this benefit was applicable to only a small proportion of the support. The majority of SND and SNP support (SND: 68.6%, SNP: 70.4%) occurred between intervener-beneficiary pairs with no observed grooming events. Furthermore, the analyses showed that only among low-ranking beneficiaries was SND and SNP support more likely in dyads that groomed frequently (>5 times), and only 2.4% (N = 19) of the total 781 instances of SND and SNP support occurred in dyads with >5 grooming events, indicating that 97.6% of SND and SNP support likely entailed no benefit toward maintaining a strong social bond and, therefore, is likely to be policing.

### Low-cost Policing Support

The *group stability hypothesis* posits that policing is low-cost for the most frequent policers, presumably the highest-ranking individuals of the group. Consistent with this prediction, high-ranking impartial interveners received return aggression from the target only once, and that aggression was of the lowest severity (a threat). SND and SNP support was also lower in cost for higher-ranking interveners. In addition, among high-rankers who intervened frequently, support of subordinate non-kin was no more costly than impartial interventions. Furthermore, as predicted, support of subordinate kin was more costly than support of subordinate non-kin (in polyadic and dyadic fights) and impartial interventions, at least among higher-ranking individuals. Notably, the pattern of increase in cost among lower-ranking individuals is quite similar to the pattern of increase in mating likelihood for both types of subordinate non-kin support (Figures 2 and 3). This suggests that as intervention cost increases for lower-ranking policers there must be a greater benefit than simply the group-level benefits of prosocial maintenance of group stability, such as increased likelihood of mating with the beneficiary.

## Conclusions

Our results show that both impartial intervention and SND and SNP support satisfy the requirements for being prosocial policing. They are (a) associated with lower group-level conflict, (b) low cost for higher-ranking interveners, and (c) frequently do not confer any additional ‘selfish’ benefits to the intervener. This is in contrast to predictions by Flack and de Waal [2004] that rhesus likely use dominance reinforcement rather than policing to maintain group stability. This prediction was likely incorrect because it was based upon the assumption that rhesus power structure is too uniform to allow for high-ranking, high-powered individuals to police others’ conflicts. However, our results show that not only do high-ranking rhesus police others’ conflicts, they do so using both impartial interventions and SND or SNP support. We propose that these partial interventions which function primarily to manage conflict be called ‘policing support interventions’. Impartial interventions alone were relatively infrequent in rhesus groups (440 total across seven groups). However, when the 408 instances of SNP support and 167 instances of SND support (those with no additional selfish benefits) are added, the total frequency of policing actions more than doubles. The fact that policing support actions were more frequent than impartial policing indicates that policing support likely plays an equally important role in conflict management as impartial interventions in rhesus macaques. Therefore, we suggest that the definition of ‘policing’ in nonhuman primates, should be expanded to include ‘policing support interventions’. Failure to include such interventions may miss important conflict management tactics that reflect mechanisms underlying the evolution of prosocial behavior in nonhuman animals and humans.

Our group-level results suggest impartial policing was directed most often at fights that lead to trauma, whereas support of non-kin in polyadic fights appeared to be directed at less intense fights resulting in severe aggression but not trauma. Intervention behavior, therefore, may be situationally dependent in rhesus macaques – impartial intervention may be most effective in certain situations, and SNP support in others. Indeed, human policing behavior is situationally dependent [32]. For example, when one person attacks another, the most appropriate policing response is to direct policing action at the attacker to get him/her to stop (partial intervention). In a bidirectional fight, policing action may be directed at both participants to stop the fight (impartial intervention). Humans also tend to use non-aggressive intervention behavior when conflicts show greater risk of escalation and injury [32]. The intervention patterns that we have uncovered in rhesus macaques, therefore, likely reflect how more complex policing systems could have evolved in humans.

More broadly, the fact that policing to control conflict in rhesus groups appears to occur via two different behaviors (impartial intervention, SND or SNP support) is not surprising; policing appears to be a widespread phenomenon in biology. The evolution of cooperation among subunits, be they replicating units in a cell [14], insects within a colony [13], monkeys within a social group [8], or humans within a society [1], appears to require policing to reduce conflict at the group level, particularly when overall relatedness within the group is low [14].

## Supporting Information

Table S1 The top five best-fit models of group-level severe aggression.
(DOCX)

Table S2 The top five best-fit models of group-level wounding.
(DOCX)

Table S3 The top five best-fit models of group-level social relocation.
(DOCX)

Table S4 Output for the best-fit model of intervention targeting by dominance ambiguity for polyadic fights.
(DOCX)

Table S5 Top five best fit models of intervention targeting by dominance ambiguity for polyadic fights.
(DOCX)

Table S6 Output for the best-fit model of intervention targeting by dominance ambiguity for dyadic fights.
(DOCX)

Table S7 Top five best fit models of intervention targeting by dominance ambiguity for dyadic fights.
(DOCX)

Table S8 Output for the best-fit model of mating access.
(DOCX)

Table S9 Top five best fit models of mating access.
(DOCX)

Table S10 Output for the best-fit model of support of subordinate non-kin in polyadic fights by grooming.
(DOCX)

Table S11 Top five best fit models of support of subordinate non-kin in polyadic fights by grooming.
(DOCX)

Table S12 Output for the best-fit model of support of subordinate non-kin in dyadic fights by grooming.
(DOCX)

Table S13 Top five best fit models of support of subordinate non-kin in dyadic fights by grooming.
(DOCX)

Table S14 Output for the best-fit model of policing cost.
(DOCX)

Table S15 Top five best fit models of policing cost.
(DOCX)
