# Supplementary material for: Policing in Nonhuman Primates: Partial Interventions Serve a Prosocial Conflict Management Function in Rhesus Macaques
Source: PLoS One. 2013 Oct 22;8(10):e77369. doi: 10.1371/journal.pone.0077369 (PMC3805604; doi:10.1371/journal.pone.0077369)
Supplement: Table S1 — The top five best-fit models of group-level severe aggression. (DOCX) [file pone.0077369.s001.docx]

Table S1 The top five best-fit models of group-level severe aggression

| Model predictors | AIC | Direction and significance of effect |
| --- | --- | --- |
| Kin dyadic rate, nonkin polyadic rate | 3.46 | Kin dyadic rate: (+) p = 0.007; Nonkin polyadic rate: (-) p = 0.005 |
| Dominant kin rate, nonkin polyadic rate | 9.19 | Dominant kin rate: (+) p = 0.04; Nonkin polyadic rate: (-) p = 0.03 |
| Kin dyadic rate, subordinate nonkin polyadic rate | 9.70 | Kin dyadic rate: (+) p = 0.03; Subordinate nonkin polyadic: (-) p = 0.03 |
| Kin dyadic rate, subordinate polyadic rate | 10.54 | Kin dyadic rate: (+) p = 0.05; Subordinate polyadic rate: (-) p =0.05 |
| Dominant kin rate, subordinate polyadic rate | 11.80 | Dominant kin rate: (+) p = 0.07; Subordinate polyadic rate: (-) p = 0.07 |
