# Supplementary material for: Policing in Nonhuman Primates: Partial Interventions Serve a Prosocial Conflict Management Function in Rhesus Macaques
Source: PLoS One. 2013 Oct 22;8(10):e77369. doi: 10.1371/journal.pone.0077369 (PMC3805604; doi:10.1371/journal.pone.0077369)
Supplement: Table S2 — The top five best-fit models of group-level wounding. (DOCX) [file pone.0077369.s002.docx]

Table S2 The top five best-fit models of group-level wounding

| Model predictors | AIC | Direction and significance of effect |
| --- | --- | --- |
| Dominant kin rate, impartial polyadic rate | -18.675 | Dominant kin rate: (+) p = 0.002; impartial polyadic rate: (-) p<0.001 |
| Overall aggression rate, impartial polyadic rate | -12.786 | Aggression rate: (+) p = 0.01; Impartial polyadic rate: (-) p = 0.006 |
| Kin dyadic rate, impartial polyadic rate | -10.549 | Kin dyadic rate: (+) p = 0.03; Impartial polyadic rate: (-) p = 0.009 |
| Overall aggression rate, impartial nonkin polyadic rate | -7.5622 | Aggression rate: (+) p = 0.08; Impartial nonkin polyadic: (-) p = 0.03 |
| Dominant dyadic rate, impartial polyadic rate | -5.6912 | Dominant dyadic rate: (+) p = 0.13; Impartial polyadic rate: (-) p = 0.03 |
