# Supplementary material for: Policing in Nonhuman Primates: Partial Interventions Serve a Prosocial Conflict Management Function in Rhesus Macaques
Source: PLoS One. 2013 Oct 22;8(10):e77369. doi: 10.1371/journal.pone.0077369 (PMC3805604; doi:10.1371/journal.pone.0077369)
Supplement: Table S3 — The top five best-fit models of group-level social relocation. (DOCX) [file pone.0077369.s003.docx]

Table S3 The top five best-fit models of group-level social relocation

| Model predictors | AIC | Direction and significance of effect |
| --- | --- | --- |
| Dominant dyadic rate, subordinate nonkin rate | -50.56 | Dominant dyadic rate: (+) p = 0.001; Subordinate nonkin rate: (-) p = 0.005 |
| Dominant dyadic rate, subordinate polyadic rate | -45.03 | Dominant dyadic rate: (+) p = 0.05; Subordinate polyadic rate: (-) p = 0.007 |
| Dominant dyadic rate, subordinate nonkin polyadic rate | -44.45 | Dominant dyadic rate: (+) p = 0.05; Subordinate nonkin polyadic: (-) p = 0.009 |
| Dominant kin rate, impartial polyadic rate | -40.47 | Dominant kin rate: (+) p = 0.2; Impartial polyadic rate: (-) p = 0.05 |
| Impartial polyadic rate | -39.96 | Impartial polyadic rate: (-) p = 0.02 |
