# Supplementary material for: Policing in Nonhuman Primates: Partial Interventions Serve a Prosocial Conflict Management Function in Rhesus Macaques
Source: PLoS One. 2013 Oct 22;8(10):e77369. doi: 10.1371/journal.pone.0077369 (PMC3805604; doi:10.1371/journal.pone.0077369)
Supplement: Table S4 — Output for the best-fit model of intervention targeting by dominance ambiguity for polyadic fights. (DOCX) [file pone.0077369.s004.docx]

Table S4 Output for the best-fit model of intervention targeting by dominance ambiguity for polyadic fights

|  | Coefficient | SE | p-value |
| --- | --- | --- | --- |
| Intervener sex (male) | 1.37 | 0.175 | < 0.001 |
| Intervener age (years) | 0.025 | 0.016 | 0.12 |
| Target sex (male) | 0.727 | 0.179 | < 0.001 |
| Target rank (1=highest rank) | -0.039 | 0.004 | < 0.001 |
| Target age (years) | 0.028 | 0.016 | 0.09 |
| Intervener-target dominance probability ‘d’ | 2.75 | 0.538 | < 0.001 |
| Intervener-target aggressive interaction frequency | 0.072 | 0.009 | < 0.001 |
| Intervener-target peaceful submission frequency | 0.418 | 0.171 | 0.014 |
| d × peaceful submission frequency | -0.413 | 0.177 | 0.02 |
