# Supplementary material for: Policing in Nonhuman Primates: Partial Interventions Serve a Prosocial Conflict Management Function in Rhesus Macaques
Source: PLoS One. 2013 Oct 22;8(10):e77369. doi: 10.1371/journal.pone.0077369 (PMC3805604; doi:10.1371/journal.pone.0077369)
Supplement: Table S5 — Top five best fit models of intervention targeting by dominance ambiguity for polyadic fights. (DOCX) [file pone.0077369.s005.docx]

Table S5 Top five best fit models of intervention targeting by dominance ambiguity for polyadic fights

| Model predictors | AIC | Direction and significance of effect |
| --- | --- | --- |
| Sex1, age1, sex2, rank2, age2, d, total aggression, total peaceful submission, d*peaceful submission | 2083 | Sex1: (+) p < 0.001; age1: (+) p = 0.12; sex2: (+) p < 0.001; rank2: (-) p < 0.001: age2: (+) p = 0.08; d: (+) p < 0.001; total aggression: (+) p < 0.001; peaceful submission: (+) p = 0.01; d* peaceful submission: (-) p = 0.02 |
| Sex1, age1, sex2, rank2, age2, d, total aggression | 2086 | Sex1: (+) p < 0.001; age1: (+) p = 0.14; sex2: (+) p < 0.001; rank2: (-) p < 0.001: age2: (+) p = 0.09; d: (+) p < 0.001; total aggression: (+) p < 0.001 |
| Sex1, age1, sex2, rank2, age2, d, total aggression, total peaceful submission | 2086 | Sex1: (+) p < 0.001; age1: (+) p = 0.14; sex2: (+) p < 0.001; rank2: (-) p < 0.001: age2: (+) p = 0.08; d: (+) p < 0.001; total aggression: (+) p < 0.001; peaceful submission: (+) p = 0.24 |
| Sex1, age1, sex2, rank2, age2, d, total aggression, d*age2 | 2087 | Sex1: (+) p < 0.001; age1: (+) p = 0.14; sex2: (+) p < 0.001; rank2: (-) p < 0.001: age2: (+) p = 0.08; d: (+) p < 0.001; total aggression: (+) p < 0.001; d*age2: (+) p = 0.52 |
| Sex1, rank1, sex2, rank2, d, total aggression | 2088 | Sex1: (+) p < 0.001; rank1: (-) p = 0.4; sex2: (+) p < 0.001; rank2: (-) p < 0.001; d: (+) p < 0.001; total aggression: (+) p < 0.001 |
