# Supplementary material for: Policing in Nonhuman Primates: Partial Interventions Serve a Prosocial Conflict Management Function in Rhesus Macaques
Source: PLoS One. 2013 Oct 22;8(10):e77369. doi: 10.1371/journal.pone.0077369 (PMC3805604; doi:10.1371/journal.pone.0077369)
Supplement: Table S6 — Output for the best-fit model of intervention targeting by dominance ambiguity for dyadic fights. (DOCX) [file pone.0077369.s006.docx]

Table S6 Output for the best-fit model of intervention targeting by dominance ambiguity for dyadic fights

|  | Coefficient | SE | p-value |
| --- | --- | --- | --- |
| Intervener sex (male) | 1.23 | 0.271 | <0.001 |
| Target sex (male) | 0.420 | 0.227 | 0.06 |
| Target rank (1=highest rank) | -0.032 | 0.005 | <0.001 |
| Intervener-target dominance probability ‘d’ | 2.09 | 0.801 | 0.009 |
| Intervener-target aggressive interaction frequency | 0.058 | 0.012 | <0.001 |
| Intervener-target peaceful submission frequency | 0.501 | 0.232 | 0.03 |
| d × peaceful submission frequency | -0.482 | 0.241 | 0.05 |
