# Supplementary material for: Policing in Nonhuman Primates: Partial Interventions Serve a Prosocial Conflict Management Function in Rhesus Macaques
Source: PLoS One. 2013 Oct 22;8(10):e77369. doi: 10.1371/journal.pone.0077369 (PMC3805604; doi:10.1371/journal.pone.0077369)
Supplement: Table S7 — Top five best fit models of intervention targeting by dominance ambiguity for dyadic fights. (DOCX) [file pone.0077369.s007.docx]

Table S7 Top five best fit models of intervention targeting by dominance ambiguity for dyadic fights

| Model predictors | AIC | Direction and significance of effect |
| --- | --- | --- |
| Sex1, rank1, sex2, rank2, d, total aggression, total peaceful submission, d*peaceful submission | 1357 | Sex1: (+) p < 0.001; rank1: (-) p = 0.3; sex2: (+) p = 0.07; rank2: (-) p < 0.001; d: (+) p < 0.001; total aggression: (+) p < 0.001; peaceful submission: (+) p = 0.03: d*peaceful submission: (-) p = 0.05 |
| Sex1, rank1, sex2, rank2, d, total aggression, total peaceful submission | 1359 | Sex1: (+) p < 0.001; rank1: (-) p = 0.3; sex2: (+) p = 0.07; rank2: (-) p < 0.001; d: (+) p = 0.04; total aggression: (+) p < 0.001; peaceful submission: (+) p = 0.13 |
| Sex1, rank1, sex2, rank2, d, total aggression | 1359 | Sex1: (+) p < 0.001; rank1: (-) p = 0.3; sex2: (+) p = 0.04; rank2: (-) p < 0.001; d: (+) p = 0.02; total aggression: (+) p < 0.001 |
| Sex1, rank1, sex2, rank2, age2, d, total aggression, total peaceful submission | 1360 | Sex1: (+) p < 0.001; rank1: (-) p = 0.3; sex2: (+) p = 0.06; rank2: (-) p < 0.001; age2: (+) p = 0.4; d: (+) p = 0.03; total aggression: (+) p < 0.001; peaceful submission: (+) p = 0.11 |
| Sex1, rank1, sex2, rank2, age2, d | 1389 | Sex1: (+) p < 0.001; rank1: (-) p = 0.6; sex2: (+) p < 0.001; rank2: (-) p < 0.001; age2: (+) p = 0.4; d: (+) p = 0.03 |
