# Supplementary material for: Policing in Nonhuman Primates: Partial Interventions Serve a Prosocial Conflict Management Function in Rhesus Macaques
Source: PLoS One. 2013 Oct 22;8(10):e77369. doi: 10.1371/journal.pone.0077369 (PMC3805604; doi:10.1371/journal.pone.0077369)
Supplement: Table S8 — Output for the best-fit model of mating access. (DOCX) [file pone.0077369.s008.docx]

Table S8 Output for the best-fit model of mating access

|  | Coefficient | SE | p-value |
| --- | --- | --- | --- |
| Intervener rank | -0.004 | 0.005 | 0.37 |
| Total groom | 0.83 | 0.049 | < 0.001 |
| Subord. non-kin polyadic (mating season) | 0.17 | 0.564 | 0.77 |
| Subord. non-kin dyadic (mating season) | 1.57 | 0.624 | 0.012 |
| Impartial (mating season) | 0.73 | 0.573 | 0.20 |
| Rank × Subord. non-kin polyadic (mating season) | 0.045 | 0.022 | 0.036 |
| Rank × Subord. non-kin dyadic (mating season) | 0.041 | 0.026 | 0.11 |
