# Supplementary material for: Policing in Nonhuman Primates: Partial Interventions Serve a Prosocial Conflict Management Function in Rhesus Macaques
Source: PLoS One. 2013 Oct 22;8(10):e77369. doi: 10.1371/journal.pone.0077369 (PMC3805604; doi:10.1371/journal.pone.0077369)
Supplement: Table S9 — Top five best fit models of mating access. (DOCX) [file pone.0077369.s009.docx]

Table S9 Top five best fit models of mating access

| Model predictors | AIC | Direction and significance of effect |
| --- | --- | --- |
| Intervener rank, total groom, SNP-mating season, SND-mating season, impartial-mating season, rank*SNP-mating season, rank*SND-mating season | 1730 | Intervener rank: (-) p = 0.4; total groom: (+) p < 0.001; SNP-mating season: (+) p = 0.8; SND-mating season: (+) p = 0.01; impartial-mating season: (+) p = 0.2; rank*SNP-mating season: (+) p = 0.04; rank*SND-mating season: (+) p = 0.11 |
| Intervener rank, total groom, SNP-mating season, SND-mating season, rank*SNP-mating season, rank*SND-mating season | 1730 | Intervener rank: (-) p = 0.4; total groom: (+) p < 0.001; SNP-mating season: (+) p = 0.7; SND-mating season: (+) p = 0.01; rank*SNP-mating season: (+) p = 0.04; rank*SND-mating season: (+) p = 0.14 |
| Intervener rank, intervener sex, total groom, SNP-mating season, SND-mating season, impartial-mating season, rank*SNP-mating season, rank*SND-mating season; sex*SND-mating season | 1733 | Intervener rank: (-) p = 0.4; intervener sex: (+) p = 0.6; total groom: (+) p < 0.001; SNP-mating season: (+) p = 0.6; SND-mating season: (+) p = 0.6; impartial-mating season: (+) p = 0.2; rank*SNP-mating season: (+) p = 0.04; rank*SND-mating season: (+) p = 0.10; intervener sex*SND-mating season: (+) p = 0.3 |
| Intervener rank, total groom, SNP-mating season, SND-mating season, impartial-mating season, rank*SNP-mating season | 1733 | Intervener rank: (-) p = 0.4; total groom: (+) p < 0.001; SNP-mating season: (+) p = 0.8; SND-mating season: (+) p <0.001; impartial-mating season: (+) p = 0.2; rank*SNP-mating season: (+) p = 0.03 |
| Intervener rank, total groom, SNP-mating season, impartial-mating season, rank*SNP-mating season | 1751 | Intervener rank: (-) p = 0.4; total groom: (+) p < 0.001; SNP-mating season: (+) p = 0.2; impartial-mating season: (+) p = 0.2; rank*SNP-mating season: (+) p = 0.08 |
