# Supplementary material for: Policing in Nonhuman Primates: Partial Interventions Serve a Prosocial Conflict Management Function in Rhesus Macaques
Source: PLoS One. 2013 Oct 22;8(10):e77369. doi: 10.1371/journal.pone.0077369 (PMC3805604; doi:10.1371/journal.pone.0077369)
Supplement: Table S10 — Output for the best-fit model of support of subordinate non-kin in polyadic fights by grooming. (DOCX) [file pone.0077369.s010.docx]

Table S10 Output for the best-fit model of support of subordinate non-kin in polyadic fights by grooming

|  | Coefficient | SE | p-value |
| --- | --- | --- | --- |
| Intervener sex (male) | 1.24 | 0.217 | <0.001 |
| Intervener rank | -0.025 | 0.006 | <0.001 |
| Beneficiary sex (male) | 0.172 | 0.192 | 0.37 |
| Beneficiary rank | 0.011 | 0.004 | 0.008 |
| Total groom frequency | 0.003 | 0.056 | 0.95 |
| Total interaction frequency | 0.024 | 0.008 | 0.004 |
| Beneficiary rank × total groom frequency | 0.003 | 0.001 | 0.01 |
