# Supplementary material for: Policing in Nonhuman Primates: Partial Interventions Serve a Prosocial Conflict Management Function in Rhesus Macaques
Source: PLoS One. 2013 Oct 22;8(10):e77369. doi: 10.1371/journal.pone.0077369 (PMC3805604; doi:10.1371/journal.pone.0077369)
Supplement: Table S11 — Top five best fit models of support of subordinate non-kin in polyadic fights by grooming. (DOCX) [file pone.0077369.s011.docx]

Table S11 Top five best fit models of support of subordinate non-kin in polyadic fights by grooming

| Model predictors | AIC | Direction and significance of effect |
| --- | --- | --- |
| Sex1, rank1, sex2, rank2, total groom, total interaction, rank2*total groom | 2147 | Sex1: (+) p < 0.001; rank1: (-) p < 0.001; sex2: (+) p = 0.4; rank2: (+) p < 0.001; total groom: (+) p = 0.9; total interaction: (+) p < 0.001; rank2*total groom: (+) p = 0.01 |
| Sex1, rank1, sex2, rank2, total groom, total interaction, rank1*total groom | 2150 | Sex1: (+) p < 0.001; rank1: (-) p < 0.001; sex2: (+) p = 0.4; rank2: (+) p < 0.001; total groom: (+) p = 0.1; total interaction: (+) p < 0.001; rank1*total groom: (+) p = 0.06 |
| Sex1, rank1, sex2, rank2, total groom, total interaction, rank1*total groom, rank2*total groom | 2150 | Sex1: (+) p < 0.001; rank1: (-) p < 0.001; sex2: (+) p = 0.4; rank2: (+) p < 0.001; total groom: (+) p = 0.9; total interaction: (+) p = 0.004; rank1*total groom: (+) p = 0.7; rank2*total groom: (+) p = 0.08 |
| Sex1, rank1, sex2, rank2, total groom, total interaction | 2152 | Sex1: (+) p < 0.001; rank1: (-) p < 0.001; sex2: (+) p = 0.4; rank2: (+) p < 0.001; total groom: (+) p < 0.001; total interaction: (+) p = 0.01 |
| Sex1, rank1, rank2, total groom, total interaction | 2152 | Sex1: (+) p < 0.001; rank1: (-) p < 0.001; rank2: (+) p < 0.001; total groom: (+) p < 0.001; total interaction: (+) p = 0.008 |
