# Supplementary material for: Policing in Nonhuman Primates: Partial Interventions Serve a Prosocial Conflict Management Function in Rhesus Macaques
Source: PLoS One. 2013 Oct 22;8(10):e77369. doi: 10.1371/journal.pone.0077369 (PMC3805604; doi:10.1371/journal.pone.0077369)
Supplement: Table S13 — Top five best fit models of support of subordinate non-kin in dyadic fights by grooming. (DOCX) [file pone.0077369.s013.docx]

Table S13 Top five best fit models of support of subordinate non-kin in dyadic fights by grooming

| Model predictors | AIC | Direction and significance of effect |
| --- | --- | --- |
| Sex1, rank1, sex2, rank2, total groom, total interaction, rank2*total groom | 1178 | Sex1: (+) p < 0.001; rank1: (-) p = 0.01; sex2: (+) p = 0.4; rank2: (+) p = 0.003; total groom: (-) p = 0.3; total interaction: (+) p = 0.04; rank2*total groom: (+) p = 0.004 |
| Sex1, rank1, sex2, rank2, total groom, total interaction | 1189 | Sex1: (+) p < 0.001; rank1: (-) p = 0.008; sex2: (-) p = 0.4; rank2: (+) p < 0.001; total groom: (+) p = 0.002; total interaction: (+) p = 0.11 |
| Sex1, rank1, sex2, rank2, total groom | 1190 | Sex1: (+) p < 0.001; rank1: (-) p = 0.008; sex2: (-) p = 0.5; rank2: (+) p < 0.001; total groom: (+) p < 0.001 |
| Sex1, rank1, sex2, rank2, total groom, total interaction, rank1*total groom | 1191 | Sex1: (+) p < 0.001; rank1: (-) p = 0.007; sex2: (+) p = 0.3; rank2: (+) p < 0.001; total groom: (+) p = 0.03; total interaction: (+) p = 0.10; rank1*total groom: (+) p = 0.4 |
| Sex1, rank1, sex2, rank2, total groom, total interaction, sex1*total groom | 1191 | Sex1: (+) p < 0.001; rank1: (-) p = 0.008; sex2: (+) p = 0.3; rank2: (+) p < 0.001; total groom: (+) p = 0.03; total interaction: (+) p = 0.12; sex1*total groom: (-) p = 0.6 |
