# Supplementary material for: Policing in Nonhuman Primates: Partial Interventions Serve a Prosocial Conflict Management Function in Rhesus Macaques
Source: PLoS One. 2013 Oct 22;8(10):e77369. doi: 10.1371/journal.pone.0077369 (PMC3805604; doi:10.1371/journal.pone.0077369)
Supplement: Table S14 — Output for the best-fit model of policing cost. (DOCX) [file pone.0077369.s014.docx]

Table S14 Output for the best-fit model of policing cost

|  | Coefficient | SE | p-value |
| --- | --- | --- | --- |
| Rank | -0.008 | 0.041 | 0.85 |
| Sex (male) | -0.61 | 0.220 | 0.005 |
| Intervention frequency | 0.26 | 0.106 | 0.014 |
| Type (sub nonkin dyad) | 5.60 | 2.326 | 0.016 |
| Type (sub nonkin poly) | 5.23 | 2.318 | 0.024 |
| Type (sub kin dyad) | 7.17 | 2.313 | 0.002 |
| Freq*Type (sub nonkin dyad) | -0.24 | 0.123 | 0.051 |
| Freq*Type (sub nonkin poly) | -0.14 | 0.108 | 0.18 |
| Freq*Type (sub kin dyad) | -0.16 | 0.142 | 0.26 |
| Rank*Type (sub nonkin dyad) | 0.013 | 0.041 | 0.74 |
| Rank*Type (sub nonkin poly) | 0.021 | 0.041 | 0.61 |
| Rank*Type (sub kin dyad) | 0.019 | 0.041 | 0.64 |
| Rank*Frequency | 0.037 | 0.015 | 0.015 |
| Rank*Freq*Type (sub nonkin dyad) | -0.026 | 0.016 | 0.11 |
| Rank*Freq*Type (sub nonkin poly) | -0.030 | 0.015 | 0.051 |
| Rank*Freq*Type (sub kin dyad) | -0.037 | 0.015 | 0.018 |
