# Supplementary material for: Policing in Nonhuman Primates: Partial Interventions Serve a Prosocial Conflict Management Function in Rhesus Macaques
Source: PLoS One. 2013 Oct 22;8(10):e77369. doi: 10.1371/journal.pone.0077369 (PMC3805604; doi:10.1371/journal.pone.0077369)
Supplement: Table S15 — Top five best fit models of policing cost. (DOCX) [file pone.0077369.s015.docx]

Table S15 Top five best fit models of policing cost

| Model predictors | AIC | Direction and significance of effect |
| --- | --- | --- |
| Sex, rank, intervention frequency, type (SND, SNP, or SKD), frequency*type*rank | 880.8 | Sex: (-) p = 0.005; rank: (-) p = 0.8; frequency: (+) p = 0.01; type: (all +) p < 0.03; frequency*type: (all -) p < 0.05 for SND only; rank*type: (+) p = 0.6 for all; rank*frequency: (+) p = 0.01; freq*rank*type: (-) p < 0.11 for all |
| Sex, rank, intervention frequency, type (SND, SNP, or SKD) | 884.7 | Sex: (+) p < 0.001; rank: (-) p = 0.003; frequency: (+) p < 0.001; type: (all +) p < 0.001 for all |
| Sex, rank, intervention frequency, type (SND, SNP, or SKD), frequency*type | 885 | Sex: (-) p = 0.005; rank: (+) p < 0.001; frequency: (+) p < 0.001; type: (all +) p < 0.001; frequency*type: (all -) p < 0.13 for all |
| Sex, rank, intervention frequency, type (SND, SNP, or SKD), rank*type | 887.7 | Sex: (-) p = 0.003; rank: (+) p < 0.001; frequency: (+) p < 0.001; type: (all +) p < 0.01; rank*type: (+) p > 0.5 for all |
| Rank, intervention frequency, type (SND, SNP, or SKD) | 891.9 | rank: (+) p < 0.001; frequency: (+) p < 0.001; type: (all +) p < 0.001 |
